# Notes and News

**Published:** 1893-12-30

**Authors:** 


### Notes and News.

Collected and Communicated.

We learn from America that a hospital is shortly to be opened at Staten Island. The project has been arranged by a number of ladies, and physicians resident on the island have consented to compose its medical staff.

Hospital Saturday and Sunday at Manchester have not been so successful this year, and show, indeed, a considerable falling of, amounting to £375. This will no doubt be learnt with regret by the inhabitants of the city, and the determination to retrieve this retrograde step will, we hope, result next year in the largest collection yet recorded.

The Beckenham District Fever Hospital is at present under the displeasure of the neighbourhood. The statement made against the hospital by the Local Board is that eleven recurrences of scarlet fever have resulted in patients discharged by the institution. The general feeling is that patients are discharged improperly cured, thereby running double risk for themselves and others.

We do not think that the slightly diminished support accorded to the Destitute Children's Dinners Society, as shown in their last report, need be regarded as showing any less sympathy in the object for which it is constituted. The real cause we believe to be the spread of the movement in so many directions that the one central original society naturally suffers, though very slightly. Through it 269,274 dinners were provided last year, and the funds received amounted to £1,497 10s. 5d.

Dr. Richard Dean, the Medical Director of the American Navy, has been investigating field hospital service in Europe for the last six months. He has just returned to America. He reports that Germany alone has a completely organized hospital corps. Italy is showing signs of speedy advancement in this direction, but the services of neither England nor France are in the condition that they should be. With a view to providing more suitable and speedy transport of the wounded from the battle-field, an American contemporarary suggests that tricycle gear should be fixed to the ambulance.

It is not to be wondered at that Mr. Cohen should ask the President of the Local Government Board whether he was aware of the great difficulties experienced by the Metropolitan Asylums Board in obtaining the site for a convalescent fever hospital in particular, and sites generally for fever hospitals. He also wanted to know how the Asylums Board were influenced to a decision in such cases. A great many people want to know why these long-delayed decisions should be allowed to interfere with the proper provision of accommodation for fever patients in the metropolis. Mr. Fowler's reply is not calculated to set at rest the doubts of inquirers. He practically focussed the matter of refusal on to the Tooting Common project, and said that in this and other cases the choice of alternative sites had been assented to, and that local interests had to be fully considered. So the matter rests much where it was before.

Many of our readers may have noted that omnibuses in London are all carrying knots of dark blue and yellow ribbands. These ribbands are borne in token of recognition of a kindly and generous thought, we were told, on inquiring of an omnibus driver. At a time when private and public claims make so many demands on a purse always freely opened, Lord Rothschild has not forgotten these useful servants of the public, and has presented each omnibus conductor and driver in London with a brace of pheasants, whilst those omnibuses which pass 148, Piccadilly, come in for further favours. Donations to this class shows not only a generosity but a thoughtfulness on the part of Lord Rothschild which all might emulate, for omnibus conductors and drivers are of necessity debarred from the tips which fall to the lot of most other employés at this season.

In the City of Dundee a Hospital for the Sick Poor has just been opened. This hospital is not a voluntary institution, but the project of the city authorities. In other words, it is under the administration of the Poor-Law. Nevertheless, very great general interest is shown towards it, and the apathy and apartness with which such undertakings are frequently viewed over the border are wholly absent amongst the population of Dundee. The light of public opinion will freely penetrate its walls, and a laudable desire that the sick shall be done the best by animates all concerned in its administration. In England it is an almost accepted fact that Poor-Law infirmaries cannot be expected to reach the same standard of efficiency, with a few exceptions, such as at Birmingham, as the voluntary hospitals. Dundee seems to recognise no such limitations. The Hospital for the Sick Poor claims to have been erected and arranged on the most approved modern principles. Great care has been exercised to provide the best systems of lighting, heating, and ventilation. Infectious cases are separately provided for, and every provision has been made for the disinfection of clothes and bedding. Not least important in the general arrangements are those of the nursing department. The importance of skilled nursing has been fully recognised, and the institution will take its place amongst the training schools for nursing in the country. The staff will commence with eight certificated and four probationer nurses. The hospital, provided to receive 260 patients, was built at a cost of £40,000.

